# Supplementary material for: Plasma-activated water: Mechanism and treatment duration for postharvest disease control and shelf-life enhancement of mango under ambient storage
Source: PLoS One. 2026 Apr 23;21(4):e0347546. doi: 10.1371/journal.pone.0347546 (PMC13105357; doi:10.1371/journal.pone.0347546)
Supplement: S1 Appendix — (DOCX) [file pone.0347546.s001.docx]

S1 Appendix. **Effect of PAW treatments on incidence (%) of anthracnose of mango var. Khirsapat and Fazlee upto 10 days of storage, replication, mean value, standard error.**

| **Treatment** | **Khirsapat** | | | | | **Fazlee** | | | | |
| --- | --- | --- | --- | --- | --- | --- | --- | --- | --- | --- |
|  | **Disease incidence of anthracnose** | | | | | **Disease incidence of anthracnose** | | | | |
|  | **6^th^ day** | **7^th^ day** | **8^th^ day** | **9^th^ day** | **10^th^day** | **6^th^ day** | **7^th^day** | **8^th^ day** | **9^th^ day** | **10^th^day** |
| **T_0_** | 15 | 16 | 38 | 79 | 82 | 17 | 24 | 25.44 | 59 | 88 |
| **T_0_** | 17 | 18 | 40 | 80 | 84 | 20 | 26 | 28.33 | 60 | 89 |
| **T_0_** | 19 | 20 | 42 | 81 | 86 | 25 | 28 | 31.22 | 61 | 90 |
| **Mean value**± SE* | 17±1.15 | 18±1.15 | 40±1.15 | 80±0.58 | 84±1.73 | 20±2.31 | 26±1.15 | 28.33±1.67 | 60±0.58 | 89±0.58 |
| **T_1_** | 0 | 0 | 0 | 17 | 18 | 0 | 0 | 0 | 19 | 20.01 |
| **T_1_** | 0 | 0 | 0 | 18 | 20 | 0 | 0 | 0 | 20 | 22 |
| **T_1_** | 0 | 0 | 0 | 19 | 22 | 0 | 0 | 0 | 21 | 22.99 |
| **Mean value ±** SE* | 00±00 | 00±00 | 00±00 | 18±0.58 | 20±1.15 | 00±00 | 00±00 | 00±00 | 20±0.58 | 22±1.15 |
| **T_2_** | 0 | 0 | 20 | 40 | 36.33 | 20 | 21 | 23 | 24 | 40 |
| **T_2_** | 0 | 0 | 21 | 42 | 40 | 21 | 22 | 24 | 25.99 | 41.93 |
| **T_2_** | 0 | 0 | 19 | 38 | 43.67 | 19 | 20 | 22 | 22.01 | 39 |
| **Mean value** ± SE* | 00±00 | 00±00 | 20±0.58 | 40±1.15 | 40±1.73 | 20±0.58 | 21±0.58 | 23±0.58 | 24±1.15 | 40±0.88 |
| **T_3_** | 0 | 0 | 22.66 | 42 | 38.55 | 17 | 20 | 20.82 | 38.01 | 39 |
| **T_3_** | 0 | 0 | 20.66 | 40 | 41 | 20 | 22 | 23.33 | 40 | 40 |
| **T_3_** | 0 | 0 | 18.66 | 38 | 43.45 | 23 | 24 | 25.84 | 41.99 | 41 |
| **Mean value** ± SE* | 00±00 | 00±00 | 20.66±1.15 | 40±1.15 | 41±1.15 | 20±1.73 | 22±1.15 | 23.33±1.45 | 40±1.15 | 40±0.58 |

SE*= Standard Error
