# Supplementary material for: Plasma-activated water: Mechanism and treatment duration for postharvest disease control and shelf-life enhancement of mango under ambient storage
Source: PLoS One. 2026 Apr 23;21(4):e0347546. doi: 10.1371/journal.pone.0347546 (PMC13105357; doi:10.1371/journal.pone.0347546)
Supplement: S2 Appendix — (DOCX) [file pone.0347546.s002.docx]

S2 Appendix. **Effect of PAW treatments on severity of anthracnose mango var. Khirsapat and Fazlee upto 10 days of storage. replication, mean value, standard error.**

| **Treatment** | **Khirsapat** | | | | | **Fazlee** | | | | |
| --- | --- | --- | --- | --- | --- | --- | --- | --- | --- | --- |
|  | **Disease severity of anthracnose** | | | | | **Disease severity of anthracnose** | | | | |
|  | **6^th^day** | **7^th^ day** | **8^th^ day** | **9^th^ day** | **10^th^day** | **6^th^ day** | **7^th^ day** | **8^th^ day** | **9^th^ day** | **10^th^day** |
| **T_0_** | 0.37 | 5 | 10 | 31.78 | 49.09 | 5.44 | 10.71 | 20 | 25.76 | 41.4 |
| **T_0_** | 3 | 10 | 20 | 43.33 | 61.67 | 8.33 | 18.33 | 30 | 38.33 | 56.67 |
| **T_0_** | 5.63 | 15 | 30 | 54.88 | 74.25 | 11.22 | 25.95 | 40 | 50.9 | 71.94 |
| **Mean value ±** SE* | 3.0±1.52 | 10±2.88 | 20a±5.78 | 43.33±6.67 | 61.67±7.26 | 8.33±1.67 | 18.33±4.4 | 30±5.77 | 38.33±7.26 | 56.67±8.82 |
| **T_1_** | 0 | 0 | 0 | 0.6 | 0.03 | 0 | 0 | 0 | 0.005 | 1.37 |
| **T_1_** | 0 | 0 | 0 | 2 | 2.33 | 0 | 0 | 0 | 0.23 | 3.67 |
| **T_1_** | 0 | 0 | 0 | 4.6 | 4.63 | 0 | 0 | 0 | 0.455 | 5.97 |
| **Mean value** ± SE* | 0.0±0.0 | 0.0±00 | 0.0±0.0 | 2.00±1.50 | 2.33±1.33 | 0.0±0.0 | 0.0±0.0 | 0.0±0.0 | 0.23±0.13 | 3.67±1.33c |
| **T_2_** | 0 | 0 | 0.6 | 5.44 | 10 | 0.06 | 0.59 | 5.25 | 10.44 | 15.44 |
| **T_2_** | 0 | 0 | 2 | 8.33 | 15 | 0.27 | 2.67 | 7.33 | 13.33 | 18.33 |
| **T_2_** | 0 | 0 | 4.6 | 11.22 | 20 | 0.48 | 4.75 | 9.41 | 16.22 | 21.22 |
| **Mean value** ± SE* | 0.0±0.0 | 0.0±00 | 2.0±1.5 | 8.33±1.67 | 15.00±2.89 | 0.27±0.12 | 2.67±1.20 | 7.33±1.20 | 13.33±1.67 | 18.33±1.67 |
| **T_3_** | 0 | 0 | 1.37 | 4.03 | 11.10 | 0.14 | 1.37 | 5.44 | 15 | 30.9 |
| **T_3_** | 0 | 0 | 3.67 | 11.67 | 18.67 | 0.37 | 3.67 | 8.33 | 20 | 36.67 |
| **T_3_** | 0 | 0 | 5.97 | 19.31 | 35.24 | 0.6 | 5.97 | 11.22 | 25 | 42.44 |
| **Mean value** ± SE* | 0.0±0.0 | 0.0±00 | 3.67±1.33 | 11.67±4.41 | 21.67±7.26 | 0.37±0.13 | 3.67±1.33 | 8.33±1.67 | 20.00±2.89 | 36.67±3.33 |

SE*= Standard Error
