# Supplementary material for: Plasma-activated water: Mechanism and treatment duration for postharvest disease control and shelf-life enhancement of mango under ambient storage
Source: PLoS One. 2026 Apr 23;21(4):e0347546. doi: 10.1371/journal.pone.0347546 (PMC13105357; doi:10.1371/journal.pone.0347546)
Supplement: S3 Appendix — (DOCX) [file pone.0347546.s003.docx]

S3 Appendix. **Effect of PAW treatments on incidence (%) of stem-end rot, replication, mean value, standard error.**

| **Treatment** | **Khirsapat** | | | | | **Fazlee** | | | | |
| --- | --- | --- | --- | --- | --- | --- | --- | --- | --- | --- |
|  | **Disease incidence (%) of stem end rot** | | | | | **Disease incidence (%) of stem end rot** | | | | |
|  | **6^th^day** | **7^th^ day** | **8^th^ day** | **9^th^ day** | **10^th^day** | **6^th^ day** | **7^th^ day** | **8^th^ day** | **9^th^ day** | **10^th^day** |
| **T_0_** | 17 | 19.02 | 21.01 | 39.76 | 58.01 | 19 | 19.51 | 20 | 58 | 77 |
| **T_0_** | 20 | 21.33 | 23 | 40.33 | 60 | 20 | 20.67 | 25 | 60 | 80 |
| **T_0_** | 23 | 23.64 | 24.99 | 40.9 | 61.99 | 21 | 21.83 | 30 | 62 | 83 |
| **Mean value** ± SE* | 20±1.73 | 21.33±1.33 | 23±1.15 | 40.33±0.33 | 60±1.15 | 20±0.58 | 20.67±0.67 | 25±2.89 | 60±1.15 | 80.00±1.73 |
| **T_1_** | 0 | 0 | 0 | 0 | 18 | 0 | 0 | 0 | 0 | 0 |
| **T_1_** | 0 | 0 | 0 | 0 | 20 | 0 | 0 | 0 | 0 | 0 |
| **T_1_** | 0 | 0 | 0 | 0 | 22 | 0 | 0 | 0 | 0 | 0 |
| **Mean value** ± SE* | 0.0±0.0 | 0.0±0.0b | 0.0±0.0 | 0.0±0.0 | 20±1.15 | 0.0±0.0 | 0.0±0.0 | 0.0±0.0 | 0.0±0.0 | 0.0±0.0 |
| **T_2_** | 0 | 0 | 0 | 19 | 20 | 0 | 0 | 0 | 19 | 19.76 |
| **T_2_** | 0 | 0 | 0 | 20 | 21 | 0 | 0 | 0 | 20 | 20.33 |
| **T_2_** | 0 | 0 | 0 | 21 | 22 | 0 | 0 | 0 | 21 | 20.9 |
| **Mean value** ± SE* | 0.0±00 | 0.0±0.0 | 0.0±0.0 | 20±0.58 | 21±0.58 | 0.0±0.0 | 0.0±0.0 | 0.0±0.0 | 0.0±00 | 0.0±0.0 |
| **T_3_** | 0 | 18.01 | 18.66 | 19.51 | 30 | 18.15 | 18 | 18.59 | 29 | 39 |
| **T_3_** | 0 | 20 | 20.33 | 20.67 | 31 | 19.67 | 20 | 20.67 | 30 | 40 |
| **T_3_** | 0 | 21.99 | 22 | 21.83 | 32 | 21.19 | 22 | 22.75 | 31 | 41 |
| **Mean value** ± SE* | 0.0±0.0 | 20±1.15 | 20.33±0.88 | 20.67±0.67 | 30±0.58 | 19.67±0.88 | 20±1.15 | 20.67±1.20 | 30±0.58 | 40.00±0.58 |

SE*= Standard Error
