# Supplementary material for: Plasma-activated water: Mechanism and treatment duration for postharvest disease control and shelf-life enhancement of mango under ambient storage
Source: PLoS One. 2026 Apr 23;21(4):e0347546. doi: 10.1371/journal.pone.0347546 (PMC13105357; doi:10.1371/journal.pone.0347546)
Supplement: S4 Appendix — (DOCX) [file pone.0347546.s004.docx]

S4 Appendix. **Effect of PAW treatments on severity (%) of stem end rot of mango var. Khirsapat and Fazlee, replication, mean value, standard error.**

| **Treatment** | **Khirsapat** | | | | | **Fazlee** | | | | |
| --- | --- | --- | --- | --- | --- | --- | --- | --- | --- | --- |
|  | **Disease severity (%) of stem end rot** | | | | | **Disease severity (%) of stem end rot** | | | | |
|  | **6^th^day** | **7^th^ day** | **8^th^ day** | **9^th^ day** | **10^th^day** | **6^th^ day** | **7^th^ day** | **8^th^ day** | **9^th^ day** | **10^th^day** |
| **T_0_** | 3.17 | 30 | 49.03 | 70.69 | 80 | 10 | 20 | 41.26 | 49.1 | 87.56 |
| **T_0_** | 4.33 | 40 | 56.67 | 78.33 | 90 | 15 | 30 | 51.67 | 61.67 | 93.33 |
| **T_0_** | 5.49 | 50 | 64.31 | 85.97 | 100 | 20 | 40 | 62.08 | 74.24 | 99.1 |
| **Mean value** ± SE* | 4.33±0.67 | 40±5.77 | 56.67±4.41 | 78.33±4.41 | 90.00±5.77 | 15.00±2.89 | 30.00±5.77 | 51.67±6.01 | 61.67±7.26 | 93.33±3.33 |
| **T_1_** | 0 | 0 | 0 | 0 | 10 | 0 | 0 | 0 | 0 | 0 |
| **T_1_** | 0 | 0 | 0 | 0 | 15 | 0 | 0 | 0 | 0 | 0 |
| **T_1_** | 0 | 0 | 0 | 0 | 20 | 0 | 0 | 0 | 0 | 0 |
| **Mean value** ± SE* | 0.0±0.0 | 0.0±0.0 | 0.0±0.0 | 0.0±00 | 15.00±2.88 | 0.0±0.0 | 0.0±0.0 | 0.0±0.0 | 0.0±0.0 | 0.0±0.0 |
| **T_2_** | 0 | 0 | 0 | 10 | 19.76 | 0 | 0 | 0 | 2.27 | 5.44 |
| **T_2_** | 0 | 0 | 0 | 20 | 20.33 | 0 | 0 | 0 | 4 | 8.33 |
| **T_2_** | 0 | 0 | 0 | 30 | 20.9 | 0 | 0 | 0 | 5.73 | 11.22 |
| **Mean value** ± SE* | 0.0±0.0 | 0.0±0.0 | 0.0±0.0 | 20.00±5.77 | 20.33±0.33 | 0.0±0.0 | 0.0±0.0 | 0.0±0.0 | 4.0±1.0 | 8.33±1.67 |
| **T_3_** | 0 | 5 | 5 | 30 | 60 | 5 | 10.9 | 21.79 | 30.76 | 50 |
| **T_3_** | 0 | 5 | 10 | 40 | 70 | 5 | 16.67 | 35 | 43.33 | 60 |
| **T_3_** | 0 | 5 | 15 | 50 | 80 | 5 | 22.44 | 48.21 | 55.9 | 70 |
| **Mean value** ± SE* | 0.0±0.0 | 5.0±0.0 | 10.00±2.89 | 40.00±5.77 | 70.00±5.77 | 5.0±0.0 | 16.67±3.33 | 35±7.63 | 43.33±7.26 | 60.00±5.77 |

SE*= Standard Error
