# Supplementary material for: Plasma-activated water: Mechanism and treatment duration for postharvest disease control and shelf-life enhancement of mango under ambient storage
Source: PLoS One. 2026 Apr 23;21(4):e0347546. doi: 10.1371/journal.pone.0347546 (PMC13105357; doi:10.1371/journal.pone.0347546)
Supplement: S5 Appendix — (DOCX) [file pone.0347546.s005.docx]

S5 Appendix**. Physiological weight loss (%) and Moisture content (%), replication, mean value, standard error.**

| **Treatment** | **Physiological weight loss (%)** | | | | **Moisture content (%)** | | | |
| --- | --- | --- | --- | --- | --- | --- | --- | --- |
|  | Khirsapat | | Fazlee | | Khirsapat | | Fazlee | |
|  | Replica-tion value | Mean value± standard error | Raw data | Mean value± standard error | Raw data | Mean value± standard error | Raw data | Mean value ± standard error |
| **T_0_** | 7.50 | 7.69±0.10 | 13.69 | 13.19±0.55 | 73.01 | 72.60±0.31 | 80.24 | 80.26±0.33 |
| **T_0_** | 7.80 |  | 12.09 |  | 72.80 |  | 79.69 |  |
| **T_0_** | 7.80 |  | 13.80 |  | 71.99 |  | 80.84 |  |
| **T_1_** | 3.09 | 3.12±0.05 | 8.82 | 8.36±0.49 | 76.79 | 77.82±0.61 | 85.34 | 85.34±0.26 |
| **T_1_** | 3.05 |  | 7.38 |  | 77.80 |  | 84.89 |  |
| **T_1_** | 3.22 |  | 8.91 |  | 78.89 |  | 85.79 |  |
| **T_2_** | 4.29 | 4.28±0.00 | 9.00 | 9.12±0.06 | 77.71 | 76.81±0.52 | 84.39 | 84.22±0.26 |
| **T_2_** | 4.29 |  | 9.20 |  | 76.83 |  | 83.72 |  |
| **T_2_** | 4.28 |  | 9.17 |  | 75.90 |  | 84.56 |  |
| **T_3_** | 5.10 | 5.22±0.06 | 11.07 | 11.54±0.40 | 75.82 | 75.32±0.46 | 82.59 | 82.89±0.51 |
| **T_3_** | 5.34 |  | 12.34 |  | 75.74 |  | 82.20 |  |
| **T_3_** | 5.23 |  | 11.22 |  | 74.40 |  | 83.88 |  |
| **Level of significance** |  | *** |  | *** |  | *** |  | *** |
