# Supplementary material for: Plasma-activated water: Mechanism and treatment duration for postharvest disease control and shelf-life enhancement of mango under ambient storage
Source: PLoS One. 2026 Apr 23;21(4):e0347546. doi: 10.1371/journal.pone.0347546 (PMC13105357; doi:10.1371/journal.pone.0347546)
Supplement: S6 Appendix — (DOCX) [file pone.0347546.s006.docx]

S6 Appendix**. P^H^ and total soluble solids (TSS), replication, mean value, standard error.**

| **Treatment** | **P^H^** | | | | **TSS** (%) | | | |
| --- | --- | --- | --- | --- | --- | --- | --- | --- |
|  | Khirsapat | | Fazlee | | Khirsapat | | Fazlee | |
|  | Replica-tion value | Mean value ± standard error | Replica-tion value | Mean value ± standard error | Replica-tion value | Mean value ± standard error | Replica-tion value | Mean value ± standard error |
| **T_0_** | 4.33 | 4.9±0.33 | 4.39 | 4.58±0.1 | 11.50 | 12.50± 0.58 | 12.50 | 14.50± 1.15 |
| **T_0_** | 4.90 |  | 4.58 |  | 12.50 |  | 14.50 |  |
| **T_0_** | 5.47 |  | 4.77 |  | 13.50 |  | 16.50 |  |
| **T_1_** | 6.41 | 6.63±0.13 | 6.25 | 6.51±0.15 | 17.00 | 18.00 ±0 .58 | 18.50 | 19.00± 0.29 |
| **T_1_** | 6.63 |  | 6.51 |  | 18.00 |  | 19.00 |  |
| **T_1_** | 6.86 |  | 6.77 |  | 19.00 |  | 19.50 |  |
| **T_2_** | 5.11 | 5.44±0.19 | 6.15 | 6.17±0.01 | 16.00 | 17.00 ± 0.58 | 16.00 | 17.00± 0.58 |
| **T_2_** | 5.44 |  | 6.17 |  | 17.00 |  | 17.00 |  |
| **T_2_** | 5.77 |  | 6.19 |  | 18.00 |  | 18.00 |  |
| **T_3_** | 5.25 | 5.44±0.11 | 5.24 | 5.53±0.17 | 17.00 | 18.00 ± 0.58 | 15.50 | 16.50± 0.58 |
| **T_3_** | 5.44 |  | 5.53 |  | 18.00 |  | 16.50 |  |
| **T_3_** | 5.63 |  | 5.82 |  | 19.00 |  | 17.50 |  |
| **Level of significance** |  | *** |  | *** |  | *** |  | ** |
