# Supplementary material for: Plasma-activated water: Mechanism and treatment duration for postharvest disease control and shelf-life enhancement of mango under ambient storage
Source: PLoS One. 2026 Apr 23;21(4):e0347546. doi: 10.1371/journal.pone.0347546 (PMC13105357; doi:10.1371/journal.pone.0347546)
Supplement: S7 Appendix — (DOCX) [file pone.0347546.s007.docx]

S7 Appendix**. Firmness and shelf-life of mango, replication, mean value, standard error.**

| **Treatment** | **Firmness (%)** | | | | **Shelf life (day)** | | | |
| --- | --- | --- | --- | --- | --- | --- | --- | --- |
|  | Khirsapat | | Fazlee | | Khirsapat | | Fazlee | |
|  | Replica-tion value | Mean value ± standard error | Replica-tion value | Mean value ± standard error | Replica-tion value | Mean value ± standard error | Replica-tion value | Mean value ± standard error |
| **T_0_** | 6.02 | 5.76±0.19 | 6.00 | 5.79±0.15 | 4.00 | 4.00 ± 0.00 | 4.10 | 4.67±0.33 |
| **T_0_** | 5.37 |  | 5.49 |  | 4.00 |  | 4.67 |  |
| **T_0_** | 5.89 |  | 5.89 |  | 4.00 |  | 5.25 |  |
| **T_1_** | 3.15 | 4.20±0.53 | 3.13 | 6.33±0.33 | 6.00 | 6.00± 0.00 | 5.76 | 6.33±0.33 |
| **T_1_** | 4.81 |  | 4.71 |  | 6.00 |  | 6.33 |  |
| **T_1_** | 4.63 |  | 4.33 |  | 6.00 |  | 6.90 |  |
| **T_2_** | 4.39 | 4.58±0.12 | 3.39 | 5.67±0.33 | 5.00 | 5.33± 0.33 | 5.10 | 5.67±0.33 |
| **T_2_** | 4.54 |  | 4.51 |  | 6.00 |  | 5.67 |  |
| **T_2_** | 4.81 |  | 4.81 |  | 5.01 |  | 6.24 |  |
| **T_3_** | 5.00 | 4.83±0.11 | 4.90 | 5.00±0.00 | 5.00 | 5.00 ± 0.00 | 5.00 | 5.00±0.00 |
| **T_3_** | 4.89 |  | 4.79 |  | 5.00 |  | 5.00 |  |
| **T_3_** | 4.62 |  | 4.66 |  | 5.00 |  | 5.00 |  |
| **Level of significance** |  | * |  | * |  | *** |  | ** |
