# Supplementary material for: Plasma-activated water: Mechanism and treatment duration for postharvest disease control and shelf-life enhancement of mango under ambient storage
Source: PLoS One. 2026 Apr 23;21(4):e0347546. doi: 10.1371/journal.pone.0347546 (PMC13105357; doi:10.1371/journal.pone.0347546)
Supplement: S8 Appendix — (DOCX) [file pone.0347546.s008.docx]

S8 Appendix**. Mineral content of mango var. Khirsapat, replication, mean value, standard error.**

| **Treatment** | **Mineral content of mango var. Khirsapat** | | | | | | | |
| --- | --- | --- | --- | --- | --- | --- | --- | --- |
|  | Replication value | | | | Mean value ± standard error | | | |
|  | K(potassium) | Ca(calcium) | Mg(Magnesium) | P(phosphorus) | K(potassium) | Ca(calcium) | Mg(Magnesium) | P(phosphorus) |
| **T_0_** | 16.99 | 24.96 | 7.00 | 16.77 | 16.99±0.01 | 24.97±0.00^d^ | 7.00±0.00 | 16.76±0.00 |
| **T_0_** | 17.00 | 24.98 | 7.01 | 16.76 |  |  |  |  |
| **T_0_** | 16.98 | 24.97 | 7.00 | 16.76 |  |  |  |  |
| **T_1_** | 20.59 | 27.64 | 10.06 | 19.09 | 20.58 ±0.00 | 27.64±0.00^a^ | 10.05±0.03 | 19.08±0.03 |
| **T_1_** | 20.58 | 27.65 | 10.05 | 19.08 |  |  |  |  |
| **T_1_** | 20.58 | 27.64 | 10.05 | 19.08 |  |  |  |  |
| **T_2_** | 19.86 | 26.66 | 9.93 | 18.86 | 19.89±0.04 | 26.66±0.00^b^ | 9.92±0.003 | 18.86±0.0 |
| **T_2_** | 19.85 | 26.66 | 9.92 | 18.87 |  |  |  |  |
| **T_2_** | 19.96 | 26.66 | 9.93 | 18.86 |  |  |  |  |
| **T_3_** | 17.89 | 25.46 | 8.88 | 17.66 | 17.88±0.01 | 25.46±0.00^c^ | 8.87±0.00 | 17.67±0.01 |
| **T_3_** | 17.88 | 25.45 | 8.87 | 17.67 |  |  |  |  |
| **T_3_** | 17.87 | 25.46 | 8.87 | 17.68 |  |  |  |  |
| **Level of significance** |  |  |  |  | *** | *** | *** | *** |
