# Supplementary material for: Plasma-activated water: Mechanism and treatment duration for postharvest disease control and shelf-life enhancement of mango under ambient storage
Source: PLoS One. 2026 Apr 23;21(4):e0347546. doi: 10.1371/journal.pone.0347546 (PMC13105357; doi:10.1371/journal.pone.0347546)
Supplement: S9 Appendix — (DOCX) [file pone.0347546.s009.docx]

S9 Appendix**. Mineral contents of mango var. Fazlee, replication, mean value, standard error.**

| **Treatment** | **Mineral content of mango var. Fazlee** | | | | | | | |
| --- | --- | --- | --- | --- | --- | --- | --- | --- |
|  | Replication value | | | | Mean value ± standard error | | | |
|  | K(potassium) | Ca(calcium) | Mg(Magnesium) | P(phosphorus) | K(potassium) | Ca(calcium) | Mg (Magnesium) | P(phosphorus) |
| **T_0_** | 17.33 | 7.77 | 3.39 | 13.39 | 17.34±0.01 | 7.76±0.00 | 3.38±0.01 | 13.38±0.00 |
| **T_0_** | 17.34 | 7.76 | 3.38 | 13.38 |  |  |  |  |
| **T_0_** | 17.35 | 7.76 | 3.37 | 13.38 |  |  |  |  |
| **T_1_** | 18.63 | 9.87 | 5.67 | 15.77 | 18.64±0.01 | 9.87±0.00 | 5.66±0.00 | 15.78±0.01 |
| **T_1_** | 18.64 | 9.87 | 5.67 | 15.78 |  |  |  |  |
| **T_1_** | 18.65 | 9.87 | 5.66 | 15.79 |  |  |  |  |
| **T_2_** | 18.33 | 9.23 | 5.23 | 14.99 | 18.33±0.00 | 9.23±0.01 | 5.23±0.00 | 14.98±0.00 |
| **T_2_** | 18.34 | 9.23 | 5.24 | 14.98 |  |  |  |  |
| **T_2_** | 18.34 | 9.22 | 5.23 | 14.98 |  |  |  |  |
| **T_3_** | 17.94 | 9.08 | 4.64 | 14.23 | 17.95±0.01 | 9.07±0.00 | 4.64±0.00 | 14.22±0.00 |
| **T_3_** | 17.95 | 9.07 | 4.65 | 14.22 |  |  |  |  |
| **T_3_** | 17.96 | 9.07 | 4.65 | 14.22 |  |  |  |  |
| **Level of significance** |  |  |  |  | *** | *** | *** | *** |
