# Supplementary material for: Plasma-activated water: Mechanism and treatment duration for postharvest disease control and shelf-life enhancement of mango under ambient storage
Source: PLoS One. 2026 Apr 23;21(4):e0347546. doi: 10.1371/journal.pone.0347546 (PMC13105357; doi:10.1371/journal.pone.0347546)
Supplement: S10 Appendix — (DOCX) [file pone.0347546.s010.docx]

S10 Appendix**. Vitamin-C content, replication, mean value, standard error.**

| **Treatment** | **Vitamin-c ( mg/100g )** | | | |
| --- | --- | --- | --- | --- |
|  | Replication value | | Mean value ± standard error | |
|  | Khirsapat | Fazlee | Khirsapat | Fazlee |
| **T_0_** | 19.55 | 29.38 | 20.19±0.33 | 28.53±0.43 |
| **T_0_** | 20.38 | 27.98 |  |  |
| **T_0_** | 20.66 | 28.23 |  |  |
| **T_1_** | 16.58 | 25.98 | 16.07±0.39 | 25.10±0.50 |
| **T_1_** | 15.29 | 24.24 |  |  |
| **T_1_** | 16.34 | 25.09 |  |  |
| **T_2_** | 17.43 | 26.09 | 17.87±0.43 | 26.16±0.23 |
| **T_2_** | 18.73 | 25.80 |  |  |
| **T_2_** | 17.45 | 26.59 |  |  |
| **T_3_** | 18.26 | 27.02 | 18.44±0.74 | 27.04±0.17 |
| **T_3_** | 17.26 | 26.75 |  |  |
| **T_3_** | 19.79 | 27.34 |  |  |
| **Level of significance** |  |  | *** | *** |
